# Parental Embodied Mentalizing Assessment^TM^ as a function of parenting stress and emotion regulation difficulties: a comparative study between postpartum depressed and nondepressed mother-infant dyads

**DOI:** 10.1186/s12888-026-08258-9

**Published:** 2026-06-16

**Authors:** Virginia Simon, Steffen Zitzmann, Frank Vitinius, Brigitte Ramsauer

**Affiliations:** 1 Faculty of Medicine, MSH Medical School Hamburg – University of Applied Sciences and Medical University, Am Kaiserkai 1, 20457 Hamburg, Germany; 2 Department of Psychosomatic Medicine, Robert Bosch Hospital, Auerbachstraße 110, 70376 Stuttgart, Germany; 3 Faculty of Humanities, MSH Medical School Hamburg – University of Applied Sciences and Medical University, Am Kaiserkai 1, 20457 Hamburg, Germany; 4 Department of Psychosomatics and Psychotherapy, Faculty of Medicine and University Hospital Cologne, University of Cologne, Weyertal 76, 50931 Cologne, Germany; 5 Department of Child and Adolescent Psychiatry and Psychotherapy, University Hospital Hamburg-Eppendorf, UKE, Martinistraße 52, 20251 Hamburg, Germany

**Keywords:** Parental Embodied Mentalizing Assessment^TM^ (PEMA^TM^), Parenting stress, Difficulties in emotion regulation, Postpartum depression, Mother-infant interaction, Parental embodied mentalizing (PEM)

## Abstract

**Background:**

In the early preverbal mother-infant communication, the impact of postpartum depression (PPD) on Parental Embodied Mentalizing (PEM) remains unclear. PEM may also depend on maternal parenting stress and emotion regulation difficulties. This study is the first to investigate PEM in mothers with and without PPD using the clinical coding system Parental Embodied Mentalizing Assessment (PEMA^TM^). It was hypothesized that PPD-mothers exhibit more PEMA^TM^ risk and fewer protective factors as a function of higher parenting stress and greater difficulties in emotional regulation compared to controls.

**Methods:**

DSM-IV diagnosed PPD- (*n* = 68) and non-diagnosed mothers (*n* = 61) with infants aged 3–10 months were assessed during a 5-minute videotaped, free-play interaction. The observational tool PEMA^TM^ categorizes kinesthetic movement patterns into eight risk and four protective factors. Independent t-tests compared group differences. Multiple linear regressions examined the Parenting Stress Index (PSI) and Difficulties in Emotion Regulation Scale (DERS) as predictors of PEMA^TM^ factors, controlling for sociodemographic covariates.

**Results:**

Significant group differences were identified (*d* = 0.40 to 1.30). PEMA^TM^ risk factors were higher in PPD-mothers, but sociodemographic variables emerged as significant confounders. Only *Obstructing Self-Regulation* was predicted by the parenting stress child domain (ß = 0.36 to 0.37), whereas *Developmental Inadequacy* was predicted by the parenting stress parent domain (ß = −0.58 to −0.55), and, after controlling for covariates, by difficulties in emotion regulation (ß = 0.48 to 0.52). Protective factors, especially *Sustained Presence*, were significantly lower in PPD-mothers and predicted by the parenting stress child domain (ß = −0.43 to −0.32).

**Conclusions:**

All mothers exhibited mainly risk-related PEM; however, PPD-mothers demonstrated more pronounced and frequent risk factors compared to the control group. Protective factors were reduced among PPD-mothers due to elevated child-related parenting stress. The consideration of child-related parenting stress and sociodemographic risk separately, within the context of diagnostic specificity in PPD, is warranted. Further research is needed on developing clinical thresholds and understanding the impact of the PEMA™ factors on child development.

**Supplementary Information:**

The online version contains supplementary material available at 10.1186/s12888-026-08258-9.

## Introduction

Parental mentalizing is defined as the parent’s child-focused capacity to appreciate and respond to the child’s mental states [1], and has mostly been studied through explicit, verbal approaches (e.g., Parental Reflective Functioning [2]). The concept has been linked to child attachment security, self-regulation, and early agency [3, 4]. During the first year of life of the preverbal infant, the embodied communication serves as the primary medium in the early parent-infant relationship [5–7]. It is expected that the parent will mirror, respond to, and thereby regulate the infant’s affective states, which are inherently nonverbal and unconscious. In this reciprocal exchange, it is essential for the parent to concurrently regulate their own mental and affective states [8].

Shai and colleagues introduced the concept of “Parental Embodied Mentalizing” (PEM [6, 9, 10]); as an adaptation of a nonverbal, implicit framework of parental mentalizing. PEM captures how the parents recognize and adjust their own kinesthetic patterns in response to their infant’s kinesthetic cues, which are understood as embodied manifestations of their mental states. To this end, spatial and temporal movement qualities (e.g., shape, pacing) in mutual whole-body kinesthetic exchanges are observed during video-recorded parent-infant interactions [6, 9, 10]. The PEM coding system has been demonstrated to be a reliable and valid operational measure [11, 12]. A growing body of research has confirmed the predictive effect of PEM on a variety of child outcomes, such as attachment security, cognitive and social skills, emotion recognition, and mental health [11, 13–15]. Furthermore, the social-cognitive concept of parental mentalizing has previously been considered as a relatively stable maternal trait across time and contexts [1, 16]. The implicit dimension PEM appears to be more state-dependent [5]. Research conducted on non-clinical samples has demonstrated that PEM quality is positively associated with maternal age, education level, and socioeconomic status and negatively related to parenting stress [14, 15, 17]. PEM has been documented to play a protective role in the prevention of parenting stress [12].

Parenting stress is defined as the distress experienced by the parent in the interaction with the child. It depends on external factors (e.g., social support) and personal characteristics of the parent and child, as measured by the Parenting Stress Index (PSI [18]). Increased stress has been shown to impair parents’ cognitive processes and emotion regulation [5, 19, 20], and emotion regulation difficulties have been linked to parents’ difficulty understanding their child’s emotions [21, 22]. Parenting stress may hinder maternal mentalizing by shifting it from a controlled and reflective to a more automatic, reactive dimension [23–25]. The presence of persisting high parenting stress has been associated with suboptimal parenting behaviors, including diminished responsiveness and lack of warmth [26–29]. Impairments in emotion regulation may further limit the parent’s capacity for sensitive and attuned mother-infant interaction. Within the process of mentalizing, the parent is considered a mediator infants affective states [30]. Affects are defined as the automatic and implicit processes embedded in nonverbal and unconscious dimensions of emotion regulation [31–35]. Consequently, parenting stress and emotion regulation difficulties may also impede parents’ ability to attune to their own and their infant’s mental states on an implicit, embodied level. Accordingly, it can be hypothesized that impairments in PEM may be associated with parenting stress and difficulties in emotion regulation.

Chronic difficulties in emotion regulation have been identified as both a fundamental criterion and a consequence of various psychiatric disorders, resulting in significant functional impairments [36]. Symptoms of depression have demonstrated a deleterious effect on explicit mentalization and emotion regulation capacities [37, 38], in particular in the parenting context [39, 40]. Postpartum depression (PPD) is defined as a state in which clinically significant depressive symptoms emerge within the first year following childbirth. These symptoms may meet diagnostic criteria for a major depressive disorder (MDD) or other depressive disorder diagnoses [41, 42]. PPD has been linked to increased parenting stress as well as emotion dysregulation [43, 44]. Therefore, PPD appears to be of additional relevance for impairments in PEM compared to mentally healthy mothers.

For clinical application, Shai and colleagues adapted the PEM coding system into the Parental Embodied Mentalizing Assessment^TM^ (PEMA^TM^ [45]). The PEMA^TM^ is composed of twelve PEMA^TM^ factors that aim to economically categorize the mother’s movement qualities in terms of the variety and quality of the commonly observed kinesthetic movement patterns during the interaction with her child. Eight of these are classified as PEMA^TM^ risk factors, indicating lapses in PEM, whereas four are PEMA^TM^ protective factors, representing flexible responses based on the parent’s mental representation of the child. It is hypothesized that these factors reflect the embodied communication of the implicit affective mental states, which may vary as a function of parenting stress and emotion regulation. Preliminary findings on PPD-mothers have indicated distinct impairments in PEMA^TM^ factors [46].

To the best of our knowledge, no study has compared the embodied communication of PPD-mothers to a non-clinical control group of mentally healthy mothers, nor examined how the PEMA^TM^ factors are related to critical contextual factors. The objective of the present study is to investigate the relationship between parenting stress, difficulties in emotion regulation, and PEMA^TM^. Theoretical and empirical considerations [46–49] suggest that the associations between different PEMA^TM^ factors and contextual factors may vary. It was hypothesized that (1) PPD-mothers will exhibit significantly higher PEMA^TM^ risk factors (quality and frequency) and lower PEMA^TM^ protective factors (quality and frequency) in comparison to mothers of the control group, and that (2) higher levels of parenting stress and difficulties in emotion regulation in both groups will be associated with higher PEMA^TM^ risk factors (quality and frequency) and lower PEMA^TM^ protective factors (quality and frequency). Given that the PEMA^TM^ factors represent conceptually distinct dimensions, the magnitude of these associations may vary across specific factors. Further, these relationships are expected to be stronger among PPD-mothers than in the control group.

## Methods

### Participants

The clinical sample of mother-infant dyads (*n* = 68) was recruited at the outpatient unit of the Department of Child and Adolescent Psychiatry, Psychotherapy, and Psychosomatics at the University Medical Center Hamburg-Eppendorf (UKE) as a part of a randomized controlled trial (RCT) evaluating a mother-infant treatment [50]. The study was initially approved by the local ethics committee of the Medical Board of Hamburg, Germany in August 2009 (reference number PV3269) and preregistered (Current Controlled Trials ISRCTN88988596). The inclusion criteria were mothers with a current DSM-IV-Axis-I diagnosis of depression (e.g., major depression, dysthymia, or adjustment disorder with depressive symptoms), who were fluent in German language and had infants aged 3–10 months [51].

In 2024, a non-clinical control sample of mothers and their infants (*n* = 61) was recruited in family centers and mother-infant gymnastics groups in Stuttgart. The inclusion of mothers in the control group was based on the assumption that they had not sought clinical services, suggesting that the perinatal symptom burden was not sufficiently elevated to necessitate treatment. The control group was matched to the clinical sample based on infant age and sex. The study was approved by the internal ethics committee of the MSH Medical School Hamburg, University of Applied Sciences and Medical University. Written informed consent was obtained from both custodial parents. The present study was not preregistered (Clinical Trial Number: not applicable) utilized baseline data from both groups, including sociodemographic and psychological questionnaires, as well as a videotaped five-minute free-play interactions between mothers and their infants. The video sessions were conducted in two distinct contexts: a clinical setting for PPD-mothers and a home setting for the control group. Mother-infant dyads were provided with a standardized blanket and identical toys during the free-play interaction in order to minimize potential setting-related biases. The mothers were instructed to play with their children as naturally as possible, similar to their everyday interactions. Caregiving behaviors (e.g., feeding, diapering) were not permitted or coded.

### Measures

**SCID-I and -II.** For the clinical sample, the Structured Clinical Interview (SCID [52]); was used to diagnose PPD according to the criteria of the Diagnostic and Statistical Manual of Mental Disorders, Fourth Edition (DSM-IV [53]). The SCID is a semi-structured interview method with high validity and reliability (Kappa = 0.61 to 0.83 for SCID-I and = 0.70 für SCID-II [54–56]).

**BDI.** The Beck Depression Inventory-I (BDI-I [57]); is a 21-item self-report questionnaire that assesses the severity of depressive symptoms within the past week. It was used for dimensional screening in both groups. The items are rated from zero to three, and the total score ranges from 0 to 63. Clinical cut-offs indicate depressive symptoms as “no to minimal” (0–10), “mild to moderate” (11–17), and “clinically relevant” (>18). The BDI demonstrated high internal consistency for clinical populations (Cronbach’s α = 0.86 [58]). In the control group, Cronbach’s was lower due to floor effects and the non-specificity of clinical instruments (Table 1).

**Table 1:** Sample characteristics by group

	All mothers *(N* ** = 129)**			Clinical group (*n* = 68)				Control group (*n* = 61)					*p* **value**
	*M*	*SD*	Range	*M*	*SD*	Range		*M*	*SD*	Range			
Infant age (months)	6.8	1.94	3.0–10.9	6.4	1.78	3.8–10.4		7.2	2.04	3.0–10.9			0.024*
Maternal age (years)	33.2	4.88	18.4–44.3	32.0	5.71	18.4–44.3		34.5	3.29	22.1–41.9			0.002**^b^
Maternal education (years)	12.1	1.41	9–13	11.6	1.57	9–13		12.6	0.97	9–13			0.000**^b^
	***f***	**%**		***f***	**%**			***f***	**%**				
Infant gender													0.114
Male	69	53.5		41	59.4			28	45.9				
Female	60	46.5		27	39.1			33	54.1				
Income (€ per month)^a^													0.000**
<1,500	17	13.2		15	21.7			2	3.3				
1,500–3,000	35	27.1		33	47.8			2	3.3				
>3,000	74	57.4		17	24.6			57	93.5				
	***M***	***SD***	**Range**	***M***	***SD***	**Range**	**α**	***M***	***SD***	**Range**	**α**	**t**	***p*** **Value**
BDI (Total Score)	13.7	10.57	1–46	20.1	10.74	1–46	0.91	6.6	3.45	1–20	0.67		0.000*^b^
PSI Total Stress Score	243.8	47.97	154–391	263.7	50.88	157–391		221.6	32.72	154–327			0.000**^b^
PSI Child Domain	99.2	23.90	53–198	104.5	25.70	53–166	0.93	93.3	20.36	63–198	0.85		0.007**^b^
Adaptability	9.5	3.76	5–21	10.5	4.22	5–21	0.78	8.5	2.88	5–16	0.63	−3.12	0.002*^b^
Demandingness	10.5	3.29	7–23	11.4	3.79	7–23	0.79	9.6	2.30	7–18	0.54	−3.40	0.000**^b^
Mood	27.3	5.95	13–43	28.0	6.47	13–43	0.83	26.5	5.27	19–43	0.68	−1.38	0.170
Distractibility/Hyperactivity	20.4	7.82	8–40	24.8	7.21	11–40	0.69	15.7	5.29	8–32	0.58	−8.17	0.000**^b^
Acceptability	10.7	3.19	6–22	10.3	4.01	6–22	0.72	11.1	1.84	6–18	0.50	1.52	0.132^b^
Reinforces Parent	18.4	8.59	9–40	24.8	7.28	11–40	0.43	11.5	2.05	9–17	0.36	−14.21	0.000*^b^
PSI Parent Domain	146.5	29.60	91–236	159.2	31.95	95–236	0.91	132.3	18.50	91–176	0.83		0.000**^b^
Depression	13.8	3.47	8–25	14.0	4.32	8–25	0.83	13.6	2.16	10–21	0.74	−0.72	0.474^b^
Competence	24.7	6.38	13–43	26.5	7.00	13–43	0.60	22.6	4.86	14–36	0.60	−3.79	0.000**^b^
Parental Attachment	18.7	5.63	9–32	20.6	6.31	9–32	0.42	16.5	3.74	10–24	0.15	−4.58	0.000**^b^
Spouse	22.0	5.74	9–35	24.1	5.75	13–35	0.78	19.7	4.81	9–31	0.57	−4.66	0.000**
Social Isolation	23.5	8.49	9–42	28.5	7.66	14–42	0.57	18.0	5.48	9–30	0.29	−8.95	0.000**^b^
Parent Health	20.0	5.69	7–35	21.5	6.33	9–35	0.55	18.4	4.39	7–29	0.68	−3.29	0.001**^b^
Role Restriction	32.5	8.36	20–55	36.5	9.24	20–55	0.82	28.0	3.94	21–39	0.73	−6.88	0.000**^b^
DERS (global score)	92.2	26.32	48–174	105.1	26.78	48–174	0.93	77.9	16.80	50–132	0.76		0.000**^b^
Nonacceptance	16.6	3.13	11–25	17.1	3.82	11–25	0.87	16.1	2.03	12–20	0.83	−1.80	0.074^b^
Goals	43.1	10.99	22–71	48.5	10.84	27–71	0.84	37.0	7.52	22–53	0.47	−7.02	0.000**^b^
Impulse	16.9	6.26	7–30	19.6	6.10	7–30	0.73	14.1	5.11	7–30	0.37	−5.48	0.000**
Awareness	14.3	4.64	5–25	16.4	4.57	8–25	0.52	12.1	3.63	5–20	0.82	−5.75	0.000**
Strategies	13.3	5.06	6–26	15.6	4.96	8–26	0.89	10.8	3.81	6–20	0.73	−6.24	0.000**^b^
Clarity	16.2	3.74	8–26	15.7	4.00	8–26	0.84	16.7	3.41	10–25	0.79	1.48	0.143

*Notes. BDI* Beck Depression Inventory, *PSI* Parental Stress Index, *DERS* Difficulties in Emotion Regulation Scale, *Nonacceptance* Nonacceptance of Emotional Response, *Goals* Difficulties Engaging in Goal-Directed Behavior, *Impulse* Control Difficulties, *Awareness* Lack of Emotional Awareness, *Strategies* Limited Access to Emotion Regulation Strategies, *Clarity* Lack of Emotional Clarity

^a^ = three missing values

^b^Welch-test

**PSI.** The German version of the Parenting Stress Index (PSI [18, 59]); was used to assess the parents’ self-reported stress related to the parenting of children aged from 0 to 12 years in both groups. The 101-item questionnaire uses a 5-point Likert scale ranging from 1 (strongly disagree) to 5 (strongly agree). Seven parent domain subscales measure stress resulting from parent characteristics and six child domain subscales assess stress from child characteristics. A total stress score is obtained from both domains, indicating the severity of the subjective burden of parenting (cut-off: >260 [18]). In the German PSI validation sample, Cronbach’s α was 0.91 for the child domain, and 0.92 for the parent domain [60]. Cronbach’s α for the PSI domains and subscales demonstrated considerable variability in line with the validation sample, ranging from adequate to satisfactory (Table 1).

**DERS.** The Difficulties in Emotion Regulation Scale [61] is a 36-item self-report questionnaire to assess clinically relevant difficulties with emotion regulation, particularly negative emotions, in both groups. The items are rated on a 5-point Likert scale (1 = almost never, 5 = almost always) and summed up to a global score across six subscales. Higher scores indicate greater difficulties. The subscales have demonstrated adequate internal consistency (Cronbach’s α 0.80 to 0.88 [61]). In the present study, Cronbach’s α for the DERS global score and the subscales ranged from excellent to acceptable (Table 1).

**PEMA**^**TM**^. The Parental Embodied Mentalizing Assessment^TM^ (version 2020, clinical training October 2020; PEMA^TM^ [45]); is a 12-point observational tool to assess PEM between parents and their 6-month to 2-year-old children in both groups. In contrast to the PEM coding system, which provides a frame-by-frame description of the movement process, the PEMA^TM^ was developed to economically screen, monitor, and train observable embodied communication in a clinical setting. Coding is permitted during repeated viewing of a split-screen videotaped five-minute free-play interaction in a clinical or home environment. The focus is on bodily movements, while disregarding the head and deactivating the sound. Twelve PEMA^TM^ factors are identified as analysis units: The eight PEMA^TM^ risk factors comprise (1) *Hostility*, as overt expression of aggression and attack on the infant’s mind; or (2) *Teasing* as a form of covert hostility delivered in the guise of pretense, fun, playing seductive manner; (3) *Obstructing Self-Regulation* of the infant’s attempts of self-soothing actions; (4) *Developmental Inadequacy* of physical support and the parent expecting too much from the infant in relation to their developmental capacity, (5) *Objectification* of the infant as an “inanimate being” without a mind; (6) *Disembodiment* in either the parent or infant, as the decoupling from the body and mind; (7) *Control*, whereby the parent dominates and forces their mind and body on the infant in a concrete inflexible approach; or (8) *Premature Termination* of the infant’s expressions of interest and exploration. The four PEMA^TM^ protective factors include (1) *Sustained Presence* whereby the parent is actively holding back to explore open and curious about what the infant is initiating; (2) *Connectivity*, which captures an affective dyadic moment of feeling close as the parent delights in the infant and the infant enjoying that; (3) *Repair* as an error response of the parent to modify their kinesthetic patterns more accurately to the infant’s signals; and (4) *Creativity*, depicting fun and playful explorations as the parent and infant offer something unique to the interaction resulting in something unexpected and novel [45]. The parent-related PEMA^TM^ factors are rated on a 4-point Likert scale (1 = mundane interactions, 4 = striking extent), based on the child’s reaction, including the clarity of child’s intentions and the parent’s accuracy in perceiving and responding to the embodied signals [6, 9, 10]. A total score sums all PEMA^TM^ protective and risk factor incidents, considering both their quality and frequency.

The first author (V. Simon) received training from the test developers Dana Shai and Rose Spencer and was certified as reliable in coding PEMA^TM^, which requires at least 80% agreement on a non-clinical sample with the test developers. All videos were coded by the first author. The reliability of PEMA^TM^ coding was examined on randomly selected subsamples (22%) from each group. For the control group, intraclass correlation coefficients (ICCs) were calculated based on a two-way mixed effects model, single measure, absolute agreement [62] by the first author. The ICC estimates (0.89 to 0.98) indicated excellent reliability (*p* < 0.001), consistent with the interrater reliability of the clinical group, which was assessed between the first author and the test developer (R. Spencer) as the second rater (Table 2). A recent study of non-clinical fathers reported inter-rater reliability for the PEMA^TM^ scores, with ICCs ranging from 0.505 to 0.997 [63].

**Table 2:** Characteristics of intra- and interrater reliability tests

Single Measures	Clinical group^a^ *n* = 15						Control group^b^ *n* = 13					
	ICC	95% CI		F Test				95% CI		F Test		
			Value	*df1*	*df2*	*p*	ICC		Value	*df1*	*df2*	*p*
PEMA^TM^ risk factors total quality	0.946	0.849-0.981	34.70	14	14	0.000**	0.983	0.949-0.974	119.381	12	12	0.000 **
PEMA^TM^ risk factors total frequency	0.893	0.771-0.963	16.69	14	14	0.000**	0.967	0.895-0.990	54.794	12	12	0.000**
PEMA^TM^ protective factors total quality	0.980	0.910-0.995	116.62	8	8	0.000**	0.922	0.772–0.975	25.344	12	12	0.000**
PEMA^TM^ protective factors total frequency	0.905	0.535-0.979	29.15	8	8	0.000**	0.898	0.711–0.967	18.688	12	12	0.000**

*Notes. ICC* = Intra-Class Correlation, *CI =* Confidence Interval, *p* = 0.001

risk factors total quality = sum of the quality of all risk factors; risk factors total frequency = sum of the incidents of all risk factors

protective total quality = sum of the quality of all protective factors; protective total frequency = sum of the incidents of all protective factors

^a^ two-way random effects model, type: single measurement, absolute agreement

^b^ two-way mixed effects model, type: single measurement, absolute agreement

### Statistical analysis

An a priori power analysis for the multiple linear regression model using G*Power Version 3.1 [64] estimated a sample size of *N* = 89 mother-infant dyads to detect medium effect sizes (f^2^ > 0.15 [40, 65, 66]), with 95% power, α = 0.05, and 13 predictors. Descriptive and inferential analyses were conducted using the Statistical Package for the Social Sciences (IBM Corp., SPSS Statistics, version 30). Group differences in baseline characteristics and PEMA^TM^ factors were assessed using t-tests, or Welch-tests in cases of variance heterogeneity, and Fisher’s exact test. Effect sizes were reported as Cohen’s *d* for t-tests and Hedges’ *g* for Welch- tests, with values of 0.2, 0.5, and 0.8 indicating small, medium, and large effects, respectively [65]. Multiple linear regression models (forced entry) were conducted to examine associations between the predictors (PSI child/parent domain, and DERS global score) and PEMA^TM^ risk and protective factors (quality and frequency) as the dependent variable in a first step [67]. Additionally, group status (clinical/control) was entered as a dummy-coded variable and the interaction terms were computed as the products of group status and the mean-centered predictors (Model 1). In a subsequent second model (Model 2), the covariates previously conducted on non-clinical samples (i.e., maternal age, education, income [14, 15, 17]); were added as a robustness check to account for potential confounders. To address positive skewness and approximate a normal distribution, the PEMA^TM^ factors (quality and frequency) were log-transformed. Furthermore, an additional sensitivity analysis was conducted to address the heterogeneity of PPD in the full clinical sample (*n* = 68). All multiple linear regression models were re-estimated using the same set of covariates to examine whether the observed associations remained consistent including in the clinical group mothers with MDD only (*n* = 56).

## Results

### Sample characteristics

The complete sample comprised *N* = 129 Caucasian mothers and their children, representing a middle-class, urban population (Table 1). A comparison of the two groups revealed that the mothers and infants in the clinical group were significantly younger. Furthermore, the mothers had fewer years of schooling and a lower average monthly income than the control group (*p* < 0.05; Table 1).

The clinical group demonstrated significantly higher levels of clinical depressive symptoms (BDI > 18; Hautzinger et al., 1994).^1^ Additionally, the PSI (child and parent domain, total stress score) and DERS global score were also significantly higher in the clinical group.

### Group comparison of PEMA^TM^ risk and protective factors

Overall, substantially more risk (total quality and total frequency) than protective factors (total quality and total frequency) were observed in both groups (total quality: *r*_s_ = −0.707, *p* < 0.001; total frequency: *r*_s_ = −0.608, *p* < 0.001).

**Fig. 1:**
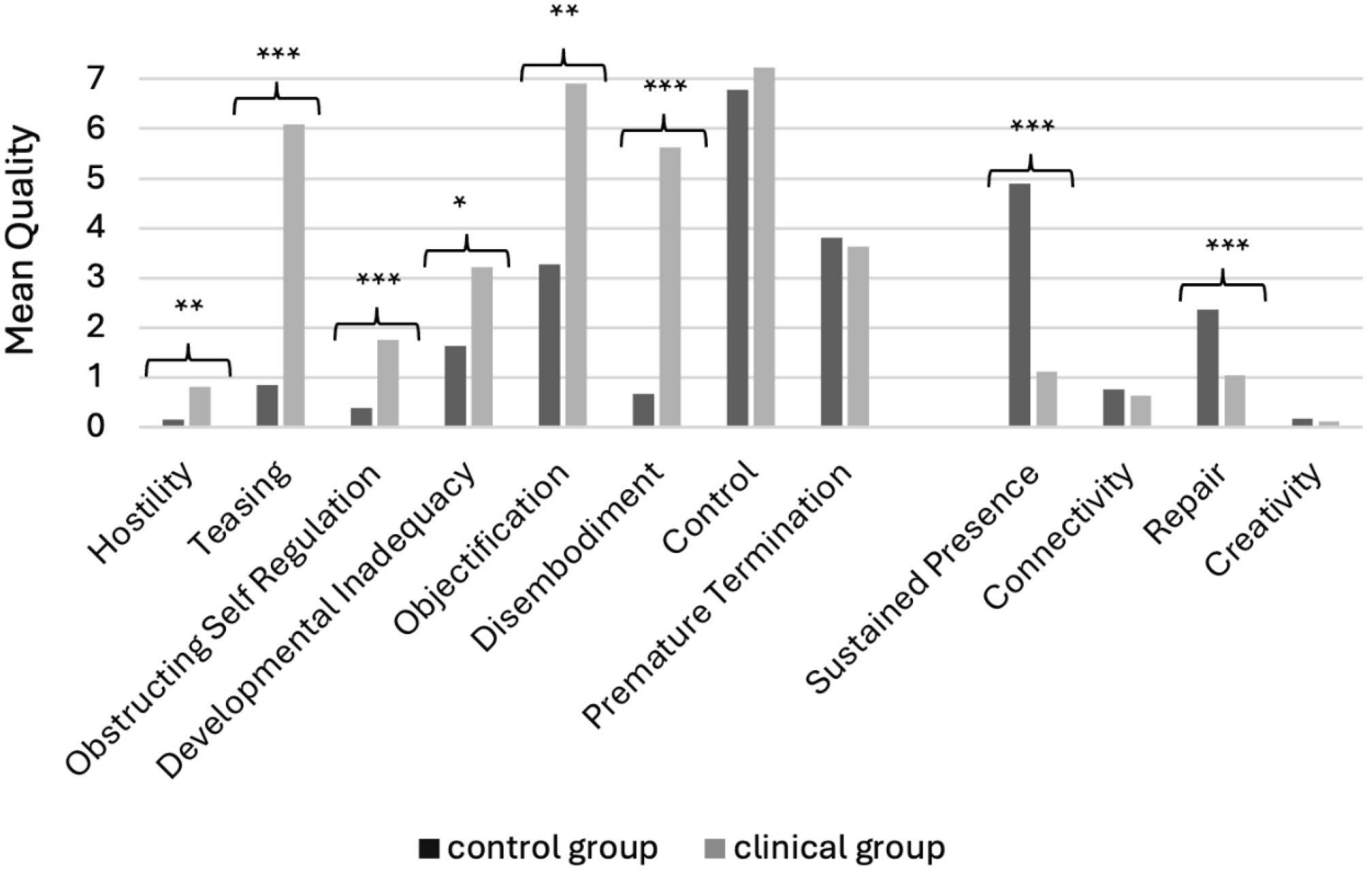
PEMA^TM^ factors mean quality. Descriptive PEMA^TM^ risk and protective factor mean quality between the clinical (*n* = 68) and control group (*n* = 61). ***p < 0.001; **p < 0.01; *p < 0.05

**Fig. 2:**
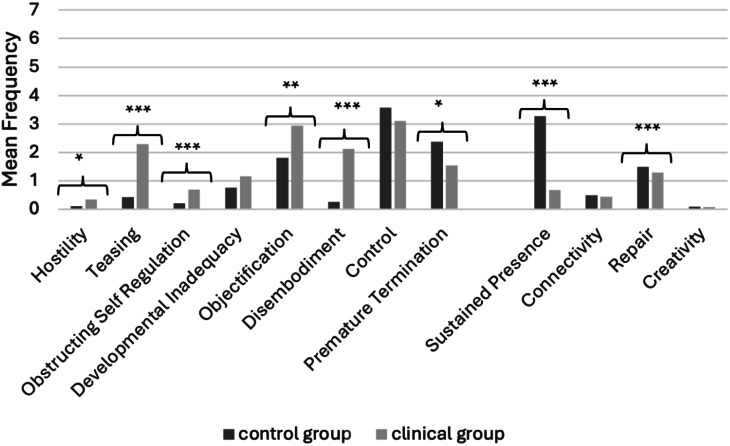
PEMA^TM^ factors mean frequency. Descriptive PEMA^TM^ risk and protective factor mean frequency between the clinical (*n* = 68) and control group (*n* = 61). ***p < 0.001; **p < 0.01; *p < 0.05

Across subgroups, the clinical group exhibited substantially more risk factors (total quality and total frequency) compared to the control group with large effect sizes. Specifically, significant group differences with large effect sizes were identified for *Teasing* (quality and frequency) and *Disembodiment* (quality and frequency). Medium effect sizes were found for *Obstructing Self-Regulation* (quality and frequency) and *Objectification* (quality and frequency). Small to medium effect sizes were identified for *Hostility* (quality and frequency) and *Developmental Inadequacy* quality. *Premature Termination* frequency was shown significantly less in the clinical group (95%-CI = [0.04, 1.59], *t*(127) = 2.08, *p*= 0.039), with a small effect size. No significant group differences were found for *Premature Termination* quality, *Developmental Inadequacy* frequency, and *Control* (quality and frequency). Additionally, the clinical group demonstrated significantly fewer protective factors (total quality and total frequency) compared to the control group with large effect sizes. Significant group differences were observed for *Sustained Presence* (quality and frequency) with large effect sizes, and *Repair* (quality and frequency) with medium to large effect sizes. *Connectivity* (quality and frequency) and *Creativity* (quality and frequency) did not differ significantly between groups (Table 3; Figs. 1 and 2).

**Table 3:** Mean differences in PEMA^TM^ factors between clinical and control group

PEMA^TM^ factors	Clinical group *n* = 68		Control group *n* = 61		*t*	*p*	effect size
	*M*	*SD*	*M*	*SD*			
Risk Factors							
Total Quality^b^	35.68	16.45	17.56	10.91	−7.44	<0.001***	
Total Frequency^a^	14.04	5.59	9.31	4.35	−5.32	<0.001***	
Hostility Quality^b^	0.81	1.79	0.15	0.48	−2.93	0.004**	0.50
Frequency^b^	0.34	0.73	0.10	0.30	−2.50	0.014*	0.42
Teasing Quality^b^	6.09	6.56	0.84	1.59	−6.40	<0.001***	1.06
Frequency	2.28	2.29	0.41	0.76	−6.37	<0.001***	1.06
Obstructing Self-Regulation Quality^b^	1.75	2.60	0.38	1.21	−3.91	<0.001***	0.67
Frequency	0.69	1.00	0.20	0.57	−3.50	<0.001***	0.60
Developmental InadequacyQuality^a^	3.22	4.78	1.62	2.84	−2.28	0.024*	0.40
Frequency^a^	1.15	1.66	0.75	1.15	−1.55	0.125	0.28
Objectification Quality^b^	6.91	6.32	3.26	4.54	−3.80	<0.001***	0.66
Frequency^a^	2.93	2.48	1.80	2.16	−2.73	0.007**	0.49
Disembodiment Quality^b^	5.63	7.90	0.66	2.84	−4.86	<0.001***	0.82
Frequency^b^	2.12	2.85	0.25	1.03	−5.07	<0.001***	0.85
Control Quality^a^	7.24	7.39	6.79	6.20	−0.37	0.711	0.07
Frequency^a^	3.10	2.79	3.56	2.75	0.93	0.354	−0.17
Premature Termination Quality^a^	3.63	4.86	3.80	4.22	0.21	0.832	−0.04
Frequency^b^	1.54	2.07	2.36	2.38	2.07	0.041*	−0.37
Protective Factors							
Total Quality^b^	2.90	4.12	8.13	6.04	5.68	<0.001**	−1.01
Total Frequency^b^	1.76	2.17	5.30	3.55	6.89	<0.001**	−1.21
Sustained Presence Quality^b^	1.10	2.43	4.89	4.22	6.15	<0.001***	−1.13
Frequency^b^	0.66	1.29	3.26	2.56	7.16	<0.001***	−1.30
Connectivity Quality^a^	0.62	1.98	0.75	1.50	0.44	0.136	−0.07
Frequency^a^	0.44	1.47	0.49	1.01	0.23	0.051	−0.04
Repair Quality^b^	1.04	1.76	2.36	2.56	3.37	<0.001***	−0.61
Frequency^b^	0.69	1.04	1.49	1.50	3.48	<0.001***	−1.11
Creativity Quality^a^	0.10	0.46	0.16	0.69	0.60	0.552	−0.10
Frequency^a^	0.06	0.24	0.08	0.33	0.46	0.647	−0.07

Notes. ***p < 0.001; **p < 0.01; *p < 0.05

risk factors total quality = sum of the quality of all risk factors; risk factors total frequency = sum of the incidents of all risk factors

protective total quality = sum of the quality of all protective factors; protective total frequency = sum of the incidents of all protective factors

^a^ standard independent t-test

^b^ Welch-test

Effect sizes for the t-tests are calculated using Cohen’s *d,* and for Welch-tests using Hedges’*g*. Negative *t*-values and effect sizes indicate that the control group showed higher mean values than the clinical group

### PEMA^TM^ factors, parenting stress, and difficulties in emotion regulation

First, the preliminary results from the initial multiple linear regression models are presented. As potential confounders were not considered, these findings should be interpreted with caution. The results were checked for robustness by running additional models, accounting for possible cofounders via the inclusion of covariates. The significant, unconfounded second models are presented in Table 4.

**Table 4:** Adjusted multiple linear regression of PEMA^TM^ factors with predictors (forced entry)

	Risk Factors					Risk Factors					Protective Factors					Protective Factors				
	Total Quality					Total Frequency					Total Quality*					Total Frequency*				
	*b*	*SE b1*	β	*t*	*p*	*b*	*SE b1*	β	*t*	*p*	*b*	*SE b1*	β	*t*	*p*	*b*	*SE b1*	β	*t*	*p*
Constant	43.39	20.05				15.11	7.12				0.81	0.58				0.53	0.48			
Group status	15.67	3.85	0.47	4.07	<0.001**	3.98	1.37	0.36	2.91	0.004*	-0.32	0.10	-0.41	-3.08	0.003*	-0.33	0.09	-0.46	-3.74	<0.001**
PSI child domain	0.17	0.10	0.24	1.70	0.091	0.07	0.04	0.29	1.96	0.052	-0.01	<0.01	-0.32	-2.16	0.034*	-0.01	<0.01	-0.43	-0.312	0.002*
PSI parent domain	-0.15	0.12	-0.27	-1.33	0.188	-0.04	0.04	-0.22	-1.00	0.319	<0.01	<0.01	0.17	0.80	0.428	<0.01	<0.01	0.28	1.45	0.152
DERS total score	0.01	0.12	0.02	0.08	0.938	<-0.01	0.04	-0.02	-0.10	0.922	<-0.01	<0.01	-0.27	-1.36	0.176	<-0.01	<0.01	-0.21	-1.11	0.271
PSI child domain*Group status	-0.14	0.13	-0.15	-1.08	0.283	-0.07	0.05	-0.24	-1.61	0.111	0.01	<0.01	0.35	2.25	0.027*	0.01	<0.01	0.36	2.48	0.015*
PSI parent domain*Group status	0.25	0.14	0.36	1.81	0.073	0.08	0.05	0.34	1.58	0.117	0.00	<0.01	-0.02	-0.08	0.934	<-0.01	<0.01	-0.05	-0.27	0.790
DERS global score*Group status	-0.07	0.15	-0.09	-0.51	0.610	-0.01	0.05	-0.05	-0.26	0.796	<0.01	<0.01	0.23	1.17	0.245	<0.01	<0.01	0.18	0.98	0.332

*Notes. SE b*^*1*^ standard error for unstandardized regression-coefficient

Risk factor total quality: *R*^2^ = 0.396 (*p* = <0.001), adjusted R^2^ = 0.333; risk factor total frequency: R^2^ = 0.309 (*p* = <0.001), adjusted R^2^ = 0.237, protective factor total quality: R^2^ = 0.367 (*p* = <0.001), adjusted R^2^ = 0.276; protective factor total frequency: R^2^ = 0.455 (*p* = <0.001), adjusted R^2^ = 0.377

To account for potential confounding, the covariates (maternal age, education, income) were statistically controlled for in the regression model

*Log-transformed data

**Table 5:** Adjusted multiple linear regression of PEMA^TM^ factors with predictors (forced entry)

	Obstructing Self-Regulation					Obstructing Self-Regulation					Developmental Inadequacy					Developmental Inadequacy				
	Quality *					Frequency *					Quality*					Frequency *				
	*b*	*SE b 1*	β	*t*	p	*b*	*SE b1*	β	*t*	*p*	*b*	*SE b1*	β	*t*	*p*	*b*	*SE b1*	β	*t*	*p*
Constant	-0.57	1.69				-0.14	1.05				1.04	2.25				0.61	1.43			
Group status	-0.05	0.66	0.39	0.82	0.945	-0.06	0.47	-0.07	-0.13	0.895	0.95	1.00	0.52	0.95	0.343	0.64	0.64	0.55	1.00	0.320
PSI child domain	0.01	0.01	0.36	2.21	0.029*	0.01	<0.01	0.37	2.25	0.026*	<0.01	0.01	0.09	0.52	0.602	<0.01	<0.01	0.13	0.75	0.457
PSI parent domain	<-0.01	0.01	-1.00	-0.42	0.679	<-0.01	<0.01	-0.07	-0.30	0.767	-0.02	0.01	-0.55	-2.26	0.026*	-0.01	0.01	-0.58	-2.34	0.021*
DERS total score	-0.01	0.01	-0.33	-1.53	0.128	-0.01	<0.01	-0.42	-1.88	0.062	0.02	0.01	0.52	2.29	0.024*	0.01	0.01	0.48	2.11	0.037*
PSI child domain*Group status	-0.01	0.01	-0.20	-1.19	0.233	<-0.01	<0.01	-0.19	-1.14	0.256	0.01	0.01	0.15	0.86	0.390	<0.01	0.01	0.12	0.68	0.498
PSI parent domain*Group status	<0.01	0.01	0.63	0.27	0.785	0.00	<0.01	0.02	0.07	0.943	0.02	0.01	0.50	2.10	0.038*	0.01	0.01	0.57	2.37	0.020*
DERS global score*Group status	0.01	0.01	0.26	1.24	0.216	0.01	<0.01	0.35	1.67	0.097	-0.02	0.01	-0.48	-2.26	0.026*	-0.01	0.01	-0.50	-2.31	0.023*

*Notes. SE b*^*1*^ standard error for unstandardized regression-coefficient

Obstructing self-regulation quality: R^2^ = 0.253 (*p* = 0.007), adjusted R^2^ = 0.139; obstructing self-regulation frequency: R^2^ = 0.238 (*p* = 0.014), adjusted R^2^ = 0.121; developmental inadequacy quality: R^2^ = 0.208 (*p* = 0.051), adjusted R^2^ = 0.086; developmental inadequacy frequency: R^2^ = 0.179 (*p* = 0.138), adjusted R^2^ = 0.054

To account for potential confounding, the covariates (maternal age, education, income) were statistically controlled for in the regression model

*Log-transformed data

**Table 6:** Adjusted multiple linear regression of PEMA^TM^ factors with predictors (forced entry)

	Sustained Presence					Sustained Presence				
	Quality *					Frequency*				
	*b*	*SE b1*	β	*t*	*p*	*b*	*SE b1*	β	*t*	*p*
Constant	0.46	1.96				0.86	1.57			
Group status	-0.13	0.87	-0.74	-1.59	0.115	-0.13	0.70	-0.82	-1.83	0.071
PSI child domain	-0.02	0.01	-0.41	-2.86	0.005**	-0.01	<0.01	-0.43	-3.08	0.003**
PSI parent domain	0.01	0.01	0.25	1.20	0.232	0.01	0.01	0.26	1.27	0.206
DERS total score	0.00	0.01	-0.01	0.05	0.960	<0.01	0.01	0.10	0.55	0.584
PSI child domain*Group status	0.01	0.01	0.28	1.93	0.057	0.01	0.01	0.29	2.04	0.044*
PSI parent domain*Group status	-0.01	0.01	-0.12	-0.57	0.568	<-0.01	0.01	-0.11	-0.58	0.564
DERS global score*Group status	<0.01	0.01	0.02	0.14	0.909	<-0.01	0.01	-0.05	-0.29	0.774

*Notes. SE b*^*1*^ standard error for unstandardized regression-coefficient

Sustained presence quality: R^2^ = 0.426 (*p* = <0.001), adjusted R^2^ = 0.338; sustained presence frequency: R^2^ = 0.457 (*p* = <0.001), adjusted R^2^ = 0.373

To account for potential confounding, the covariates (maternal age, education, income) were statistically controlled for in the regression model

*Log-transformed data

Multiple linear regression analysis revealed that neither the PSI nor the DERS, but only group status (*b* = 18.83, SE = 3.18, ß = 0.56, *p* < 0.001), showed a significant association with the risk factors total quality, and the model explained a significant proportion of variance (Model 1: *R*^2^ = 0.320, *F*(7, 121) = 8.13, *p* < 0.001). Group status remained significant after covariate adjustment and the second model explained a slightly greater proportion of variance, although no significant associations with covariates were found (Table 4). For the total frequency (Model 1: *R*^2^ = 0.219, *F*(7, 121) = 4.83, *p* < 0.001), a preliminarily significant association was found with the PSI child domain (*b* = 0.08, SE = 0.04, ß = 0.34, *p* = 0.031) as well as with group status (*b* = 4.81, SE = 1.13, ß = 0.43, *p* < 0.001). After robustness check, only the associations with group status, but not with the PSI child domain remained significant and a modestly greater amount of variance was explained (Table 4). Additionally, income below €1,500 (total quality: *b* = 15.82, SE = 6.77, *p* = 0.021; total frequency: *b* = 6.57, SE = 2.40, *p* = 0.007), between €3,000 and €5,000 (total frequency: *b* = 4.11, SE = 2.06, *p* = 0.048, and above €5,000 (total frequency: *b* = 4.96, SE = 2.27, *p* = 0.031) was found to be significantly associated.

For risk factors, the PSI child domain (*b* = 0.01, SE = 0.01, ß = 0.33, *p* = 0.043) as well as group status (*b* = 0.57, SE = 0.15, ß = 0.40, *p* < 0.001) demonstrated a significant association with *Obstructing Self-Regulation* (Model 1 for quality: *R*^2^ = 0.173, *F*(7, 121) = 3.62, *p* = 0.001; Model 1 for frequency: *R*^2^ = 0.170, *F*(7, 121) = 3.55, *p* = 0.002). The associations with the PSI child domain, but not with group status persisted after covariate adjustment (Table 4). Model 2 explained a modestly greater proportion of variance, although no significant associations with covariates were found (Table 4). The effect of the PSI child domain on *Control* quality was significantly weaker in the clinical group (*b* = −0.02, SE = 0.01, ß = −0.37, *p* = 0.037), but no variance was explained (Model 1 for quality: *R*^2^ = 0.040, *F*(7, 121) = 0.72, *p* = 0.659; Model 1 for frequency: *R*^2^ = 0.046, *F*(7, 121) = 0.84, *p* = 0.560). After robustness check, the interaction effect remained significant (Table 4) and its frequency was significantly associated with income below €1,500 (*b* = 1.89, SE = 0.88, *p* = 0.034). Despite this, still no variance was explained (Table 4).

The PSI parent domain was significantly associated with *Developmental Inadequacy* (quality: *b* = −0.02, SE = 0.01, ß = −0.51 *p* = 0.037; frequency: *b* = −0.01, SE = 0.01, ß = −0.54, *p* = 0.028) and this effect was stronger in the clinical group (quality: *b* = 0.02, SE = 0.01, ß = 0.48, *p* = 0.042; frequency: *b* = 0.01, SE = 0.01, ß = 0.54, *p* = 0.021). The preliminary model explained a significant proportion of variance (Model 1 for quality: *R*^2^ = 0.131, *F*(7, 121) = 2.61, *p* = 0.015; Model 1 for frequency: *R*^2^ = 0.118, *F*(7, 121) = 2.32, *p* = 0.030). After robustness check, the significant associations with the PSI parent domain and the interaction effect persisted (Table 4). Additionally, significant associations with the DERS global score, with a weaker effect in the clinical group (Table 4) as well as between the quality and income below €1,500 (*b* = 2.34, SE = 1.10, *p* = 0.037) emerged. However, variance was no longer explained (Table 4).

The remaining risk factors were neither associated with the PSI nor the DERS but only with group status (*Teasing* quality: *b* = 1.15, SE = 0.20, ß = 0.55, *p* = <0.001; frequency: *b* = 0.73, SE = 0.13, ß = 0.53, *p* = <0.001; *Disembodiment* quality: *b* = 1.18, SE = 0.22, ß = 0.54, *p* = <0.001; frequency: *b* = 0.79, SE = 0.15, ß = 0.53, *p* = <0.001; *Hostility* quality: *b* = 0.28, SE = 0.11, ß = 0.27, *p* = 0.015; *Hostility* frequency: *b* = 0.15, SE = 0.07, ß = 0.23; *p* = 0.039; *Objectification* quality; *b* = 0.69, SE = 0.22, ß = 0.33, *p* = 0.002; frequency: *b* = 0.43, SE = 0.16, ß = 0.29, *p* = 0.010; *Premature Termination* frequency: *b* = −0.36, SE = 0.17, ß = −0.24, *p* = 0.032). Variance was explained in the preliminary models for *Teasing (*Model 1 for quality: *R*^2^ = 0.312, *F*(7, 121) = 7.82, *p* = <0.001; Model 1 for frequency: *R*^2^ = 0.289, *F*(7, 121) = 7.03, *p* < 0.001) and *Disembodiment* (Model 1 for quality: *R*^2^ = 0.254, *F*(7, 121) = 5.90, *p* < 0.001; Model 1 for frequency: *R*^2^ = 0.253, *F*(7, 121) = 5.86, *p* < 0.001), but not for *Hostility* (Model 1 for quality: *R*^2^ = 0.092, *F*(7, 121) = 1.75, *p* = 0.105; Model 1 for frequency: *R*^2^ = 0.075, *F*(7, 121) = 1.40, *p* = 0.212), *Objectification* (Model 1 for quality: *R*^2^ = 0.121, *F*(7, 121) = 2.36, *p* = 0.260; Model 1 for frequency: *R*^2^ = 0.143, *F*(7, 121) = 1.62, *p* = 0.136), and *Premature Termination* (Model 1 for quality: *R*^2^ = 0.033, *F*(7, 121) = 0.60, *p* = 0.756; Model 1 for frequency: *R*^2^ = 0.059, *F*(7, 121) = 1.09, *p* = 0.375). After robustness check, no association with group status remained significant for the quality and frequency of *Teasing, Disembodiment, Hostility,* and *Objectification* as well as *Premature Termination* frequency (Table 4). However, a modestly greater proportion of variance was explained for the quality and frequency of *Teasing* and *Disembodiment* (Table 4). For *Teasing* frequency, the effect of mother’s age was significantly weaker (*b* = −0.07, SE = 1.00, *p* = 0.017), and the effect with mother’s school years significantly stronger in the clinical group (*b* = 0.21, SE = 1.00, *p* = 0.036). While model 2 explained a proportion of variance for *Hostility* quality, still no variance was explained for its frequency as well as the quality and frequency of *Objectification* and *Premature Termination* (Table 4). However, an additional significant association was found between *Premature Termination* and maternal age (quality: *b* = −0.09, SE = 0.04, *p* = 0.032; frequency: *b* = −0.06, SE = 0.03, *p* = 0.036), which was weaker in the clinical group (quality: *b* = −0.13, SE = 0.05, *p* = 0.005; frequency: *b* = −0.01, SE = 0.04, *p* = 0.006).

For protective factors (Model 1 for total quality: (*R*^2^ = 0.320, *F*(7,88) = 5.93, *p* < 0.001; Model 1 for total frequency: (*R*^2^ = 0.399, *F*(7,89) = 8.46, *p* < 0.001), significant associations with the PSI child domain (total quality: *b* = −0.01, SE = 0.01, ß = −0.35, *p* = 0.022; total frequency: *b* = −0.01, SE = 0.01, ß = −0.46, *p* = 0.002), with a stronger effect in the clinical group (total quality: *b* = 0.01, SE = 0.01, ß = 0.33, *p* = 0.034; total frequency: *b* = 0.01, SE = 0.01, *p* = 0.023), and with group status (total quality: *b* = −0.36, SE = 0.08, ß = −0.46, *p* = <0.001; total frequency: *b* = −0.39, SE = 0.07, ß = −0.56, *p* = <0.001) were found. The associations with the PSI child domain and the interaction effect as well as with group status remained significant after robustness check (Tables 4, 5, and 6). Model 2 explained a slightly greater proportion of variance, although no association with covariates were found (Tables 4, 5, and 6). In particular, the PSI child domain (quality: *b* = −0.02, SE = 0.01, ß = −0.40, *p* = 0.005, frequency: *b* = 0.01, SE = 0.01, ß = −0.40, *p* = 0.004), with stronger effects in the clinical group (quality: *b* = 0.01, SE = 0.01, ß = 0.29, *p* = 0.044, frequency: *b* = 0.01, SE = 0.01, ß = 0.29, *p* = 0.038), and group status (quality: *b* = −1.14, SE = 0.17, ß = −0.61, *p* = <0.001, frequency: *b* = −1.03, SE = 0.14, ß = −0.66, *p* = <0.001) were significantly associated with *Sustained Presence* (Model 1 for quality: *R*^2^ = 0.371, *F*(7, 121) = 10.21, *p* < 0.001; Model 1 for frequency: *R*^2^ = 0.402, *F*(7, 121) = 5.48, *p* < 0.001). The associations with the PSI child domain and the interaction effect for frequency but neither the interaction effect for quality nor the association with group status remained significant after robustness check (Tables 4, 5, and 6). Model 2 explained a modestly greater proportion of variance, although no significant associations with covariates emerged (Tables 4, 5, and 6).

The remaining protective factors were neither associated with the PSI nor with the DERS. No variance was explained in these models (Model 1 for *Repair* quality: *R*^2^ = 0.103, *F*(7, 121) = 1.98, *p* = 0.063; Model 1 for frequency: *R*^2^ = 0.107, *F*(7, 121) = 2.08, *p* = 0.051);Model 1 for *Connectivity* quality: *R*^2^ = 0.015, *F*(7, 121) = 0.27, *p* = 0.964; Model 1 for frequency: *R*^2^ = 0.010, *F*(7, 121) = 0.18, *p* = 0.989); Model 1 for *Creativity* quality: *R*^2^ = 0.020, *F*(7, 121) = 0.35, *p* = 0.932; Model 1 for frequency: *R*^2^ = 0.018, *F*(7, 121) = 0.33, *p* = 0.941). The preliminary association between group status and *Repair* (quality: *b* = −0.57, SE = 0.16, ß = −0.38, *p* < 0.001; frequency: *b* = −0.46, SE = 0.13, ß = −0.39, *p* < 0.001) did not remain significant after robustness check (Table 4). However, a significant association was found between its quality and mother’s school years (*b* = 0.21, SE = 0.10, *p* = 0.043), which was weaker in the clinical group (*b* = −0.28, SE = 0.12, *p* = 0.027). Still, no variance was explained in model 2 (Table 4).

Additional sensitivity analyses, restricted to PPD-mothers with MDD (*n* = 56), revealed a largely comparable overall pattern of associations with directions of effects, magnitudes, and significance remaining similar. However, after adjusting for covariates, some discrepancies emerged in this subsample in comparison to the full sample. For regression and standardized coefficients, see Additional File 1.

## Discussion

The present study investigated the embodied communication of PPD-mothers and their infants compared to a non-clinical control group, using the clinical 12-factor approach PEMA^TM^. Previous research has indicated an effect of parenting stress and difficulties in emotion regulation on the embodied communication. Consequently, these maternal psychological variables were examined as predictors of PEMA^TM^.

In general, PPD-mothers reported significantly elevated levels of parenting stress and greater difficulties in emotional regulation compared to non-clinical mothers. Further research is needed to clarify whether this heightened experience is a transient state response to the adjustment to new parental roles or a stable trait manifestation of poor intrapsychic structural integration associated with mental disorders [20, 39].

For PEMA^TM^, PPD-mothers demonstrated higher, i.e., more intense and prolonged, and frequent incidents of PEMA^TM^ risk factors than non-clinical mothers. The most prominent risk factors observed in PPD-mothers were *Objectification, Teasing,* and *Disembodiment*. These patterns of embodied communication are characterized by treating the infant like an “inanimate being”, akin to a doll, or by interactions that are subtly hostile yet disguised as playful – for example, holding a toy close to the child and pulling it away just as the infant reaches for it. Such behaviors have the potential to amplify maternal satisfaction while concurrently elevating the child’s stress levels. Furthermore, mothers exhibiting symptoms of PPD appeared more intense and often disembodied. This means that they were mentally absent, despite being physically present, and their infants tended to withdraw when distressed. Regardless of maternal mental health status, *Control* and *Premature Termination* emerged as prevalent risk factors. Protective factors were observed more frequently among non-clinical mothers, whose embodied communication was particularly characterized by more and pronounced *Sustained Presence* and *Repair*. These patterns are characterized to foster more attuned and responsive interactions with their children.

The inclusion of the hypothesized predictors provided additional explanation for the observed group differences. Given that the child’s reaction serves as a reference point for PEMA^TM^ coding, further analyses revealed that the child domain of parenting stress plays a critical role in shaping the mother’s PEM-related embodied communication. In line with these findings, prior studies on the explicit, verbal approach of parental mentalizing reported an association with maternal tolerance of infant, but not general distress [68, 69]. This study builds on previous research by examining its impact on the embodied communication in mother-infant interactions. Among the PEMA^TM^ factors examined, significant and robust relationships after controlling for potential confounders through the inclusion of covariates were identified between protective factors and the child domain of maternal parenting stress. Specifically, *Sustained Presence* was strongly predicted by maternal parenting stress related to the child domain. For risk factors, only *Obstructing Self-Regulation* showed a robust, significant association with the child domain of maternal parenting stress after accounting for covariates. In both groups, higher levels of maternal child-related parenting stress were associated with fewer as well as diminished protective factors and an increased prevalence of the risk factors *Obstructing Self-Regulation*.

For PEMA^TM^ protective factors, greater proportions of explained variance were found than for risk factors. Consequently, the present findings suggest that the mothers’ subjective experience of child-related parenting stress manifests more strongly in the reduction of an open, flexible, and attuned embodied communication with the child rather than actively induced embodied disruptions in PEM. These findings are consistent with previous studies on the relationship between parenting stress on parenting behavior [26, 27, 29]. As stress levels rise, the mother’s mental representations, which underpin her kinesthetic movement patterns, may change, potentially resulting in challenges to maintain her capacity to mentalize [69]. Previously, depressed mothers reported their children as more difficult [70]. Consistent with findings by Feldman, Granat [71] these results demonstrate, that embodied, implicit processes operate alongside, and somehow independent of explicit cognitive evaluations. Consistent with this, the interaction effect demonstrated that the child domain of maternal parenting stress had a stronger influence on protective factors in the clinical group, whereas its impact on the risk factor *Control* was stronger in the control group.

In contrast to impairments resulting from reduced PEM-related protective behavior, risk-related embodied communication was found to be less affected by parenting stress in PPD-mothers. Accordingly, *Developmental Inadequacy,* as the only PEMA^TM^ risk factor associated with the parent domain of parenting stress, was shown significantly more pronounced and frequent when the mothers reported lower subjective levels of parent-related maternal parenting stress, especially among PPD-mothers. This observation suggests that these mothers hold inappropriate, kinesthetically manifested expectations towards their children, possibly to reduce parenting stress arising from their own characteristics. Therefore, risk factors might be associated with stressors beyond parenting stress [69].

After the inclusion of covariates and thus independent of the mother’s age, education or income, greater maternal difficulties in emotion regulation appeared to contribute to developmentally excessive demands on the child. In line with these findings, difficulties in emotion regulation have previously been observed in parents as a response to stress and demanding child behavior. Sociodemographic variables have been show to play a particularly important role in shaping these associations [20]. Especially, negative parenting has been found to be associated with difficulties in emotion regulation beyond the impact of the specific psychopathology among mothers with depressive symptoms, at both a clinical and subclinical level [22, 39]. This may also explain why, contrary to our hypothesis, the impact of difficulties in emotion regulation was weaker in the clinical group. Another potential explanation for the otherwise non-significant results of the DERS may be the mediating role of more prevalent insecure attachment in PPD-mothers, which is found to be involved in the defensive suppression of emotions [72]. This emotional processing strategy could facilitate sustained other-focused mentalizing [73]. Moreover, a recent review of the framework of multidimensional mentalizing characterized the DERS as an affective, yet primarily self-focused, internally cued, and reflective measurement [74, 75]. Therefore, the conclusions derived from this approach may differ from those obtained from the child-focused, externally cued, and implicit PEMA^TM^.

In general, controlling for confounders in the robustness checks substantially altered the results for PEMA^TM^ risk factors: maternal sociodemographic characteristics had an explanatory value and confounded initial associations with the PSI and DERS on risk factor total frequency and *Developmental Inadequacy* as well as the effect of group status on *Hostility* and *Premature Termination,* and the most intensely and frequently shown risk factors *Teasing*, *Objectification,* and *Disembodiment* as well as diminished protective factors *Sustained Presence* and *Repair* among PPD-mothers. The loss of significance of these most prevalent shown PEMA^TM^ factors, particularly among PPD-mothers, suggests that the embodied communication may be more strongly associated with socioeconomic status than with depressive psychopathology per se. Certain kinesthetic movement patterns may therefore occur more intensely and frequently in contexts of socioeconomic adversity. Cross-sectional empirical studies have identified an association between socioeconomic disadvantage and harsher parental behaviors [76], and more withdrawn interactions [77]. Indeed, longitudinal epidemiological studies have shown that families experiencing social adversity experience more stress than individuals with a higher socioeconomic status [78, 79]. Lower socioeconomic status may be linked to the expression of higher risk factor expression, which warrants careful investigation in subsequent research. Future studies with at-risk samples should explicitly account for socioeconomic level, as it is highly stable and impactful factor from childhood to adulthood and even across generations [80, 81].

Several sociodemographic variables were identified as significant predictors associated with distinct PEMA^TM^ factors: lower income was linked to higher levels of risk factor total quality and frequency, *Developmental Inadequacy* quality, and *Control* frequency in both groups. Overall, the total frequency of risk-related embodied communication is not limited to the objective income level of the mothers. Additionally, younger maternal age was associated with more intense and frequent *Premature Termination.* This impact of maternal age was found to be less pronounced in PPD-mothers, similar to the association between maternal age and *Teasing* frequency. In contrast, the association between lower maternal education and increased *Teasing* frequency was stronger among PPD-mothers. However, the association between greater educational attainment and increased *Repair* quality was weaker among PPD-mothers.

These findings are consistent with studies on PEM in non-clinical samples [14, 17]: lower maternal socioeconomic status, age, and education level were generally associated with lower PEM quality, reflecting more PEMA^TM^ risk factors and fewer protective factors. Previous research has linked these sociodemographic determinants to an elevated risk for PPD onset [43, 82, 83]. The stronger effect of these sociodemographic predictors in the control group may be explained through the baseline risk in the PPD-mothers: The present clinical sample was significantly younger age, had fewer years of education, and lower income. Further research on PEMA^TM^ is needed to clarify the potential determining effect of these sociodemographic risk factors on parenting stress as well as the group status in the context of embodied communication. Future research should consider the determinants of positive and negative parenting separately, as they appear to be affected by different regulatory and contextual factors, particularly in the context of sociodemographic risk. Notably, group differences persisted for the PEMA^TM^ total scores, indicating that total scores may be more reliable than the distinct factors and therefore making significant effects easier to detect. The risk factors most prevalent in both groups, such as *Control* or *Premature Termination,* appeared to be independent of group status. Furthermore, child-related parenting stress had an even more pronounced impact in the control group. These findings suggest that these patterns may even reflect a common, non-specific response to stress or moments of dysregulation within otherwise well-functioning mother-infant interactions.

The present study raises important questions regarding the clinical relevance of the PEMA^TM^. First, independent of maternal clinical status, some degree of risk-related embodied communication was exhibited. This may support a dimensional perspective on the PEMA^TM^ factors, which could be relevant across all maternal populations. The risk factors were more intense and frequent in PPD-mothers, thus prompting a critical discussion on the need to establish a threshold for clinical relevance and intervention. Furthermore, sociodemographic risk factors may confound embodied communication independently of clinical status and must be considered. PPD-mothers appeared to have a markedly diminished ability to display protective PEMA^TM^ factors compared to non-clinical mothers, which may limit their potential to compensate for existing risk factors. For this purpose, prospective validation studies are needed to ascertain how PEMA^TM^ factors predict infant developmental outcomes in terms of quality and frequency. Since *Hostility*, *Connectivity,* and *Creativity* were rarely observed, more research is needed on their relevance to later stages of child development and across clinical groups beyond PPD-diagnosis. Second, the sensitivity analyses shed light on the role of diagnostic specificity in the context of PPD. Although the overall pattern of results remained largely consistent, the findings restricted to the subsample of PPD-mothers with MDD suggest that parenting stress has a broader impact and therefore appears to be more generally linked to PEMA^TM^ factors. This may point to an underlying depression-related mechanism that operates across diagnostic subtypes, while also indicating that some effects may depend on the diagnostic composition. Comorbidities of PPD should also be taken into account, in particular personality disorders [84]. These disorders have the potential to alter the perception, regulation strategies, defense mechanisms, and attachment [85].

### Limitations

The clinical group demonstrated excellent interrater reliability. Although comparable intrarater reliability was achieved for the control group, indicating consistent coding of the PEMA^TM^, and despite the first author’s training and certification as a reliable rater in both clinical and non-clinical contexts, the study design rendered her unblinded to group status. This is a limitation that should be considered when interpreting the results. Despite large effect sizes, floor and ceiling effects may artificially exclude observations of actual group differences beyond the measurable range of PEMA^TM^ [67]. In addition, the substantial group differences in maternal sociodemographic characteristics should be considered as a baseline risk in the clinical group, which may limit the additional explanatory power due to multicollinearity with the distinct PEMA^TM^ factors. The significance of social status as a risk factor for maternal depression has been highlighted in past theoretical and empirical work [86]. In contrast, the current control group comprises a convenience sample. Participation in the study was voluntary for ethical reasons, so that the participating mothers were highly interested and motivated in the study’s focus on early mother-infant interaction. A recent study demonstrated that convenience samples are more likely to consist of parents who are highly educated and have higher socioeconomic status than representative groups of parents [87]. A potential limitation of the study is that the control group was recruited later than the clinical group for feasibility reasons. However, the prevalence rates and key risk factors of PPD [82, 88, 89], the diagnostic criteria [53, 90], and the core construct of mother-infant interaction [91–95] have remained largely stable over time, which supports the comparability of the groups. Standardized measures (BDI, PSI, DERS) were used across both samples and including relevant sociodemographic covariates reduced potential cohort effects. However, unmeasured confounders cannot be eliminated entirely and must be considered when interpreting our results, particularly given the temporal gap between the recruitment of the clinical and control group. It should be acknowledged that clinically significant depressive symptoms in the control group cannot be ruled out entirely. Finally, the clinical group comprised a naturalistic clinical sample of PPD-mothers with clinically verified DSM-IV diagnoses of depressive disorders in the postpartum period (*n* = 68). Using standardized diagnostic strengthens the validity of our findings, while the broader spectrum of depressive disorders enhances ecological validity by capturing the heterogeneity of PPD typically observed in clinical practice. However, a further sensitivity analysis, which included only PPD-mothers with a MDD (*n* = 56), indicated that some effects may be more influenced by diagnostic heterogeneity. Importantly, restricting the analysis to a diagnostically more homogeneous subsample involves a trade-off between statistical clarity and specificity on the one hand and validity and representativeness on the other. While reduced heterogeneity may decrease variance and thereby facilitate the detection of effects, the broader clinical sample more closely reflects the diversity of PPD in research and clinical practice [42, 96–99]. The variability in statistical significance across analyses suggests that some effects may be sensitive to sample composition. Accordingly, results close to conventional significance thresholds should be interpreted with caution, as they may not reflect robust associations. Further investigation of PPD heterogeneity remain important for future research and clinical treatment. Subsequent studies with larger sample sizes could enhance the robustness and generalizability of our findings, particularly for interaction effects and the analysis of confounding variables in the context of group differences [65, 100].

## Conclusions

In summary, a higher prevalence of PEMA^TM^ risk than protective factors was observed, regardless of the mother’s clinical status. While the risk factors were more pronounced and frequent in PPD-mothers, their extent depended primarily on maternal sociodemographic characteristics (e.g., maternal age, income, and education). Only the risk factor *Obstructing Self-Regulation* emerged in relation to elevated levels of child-related parenting stress in both groups. Conversely, *Developmental Inadequacy* was associated with lower parent-related stress, particularly in the clinical group, and with greater maternal difficulties in emotion regulation after controlling for covariates. Non-clinical mothers demonstrated pronounced and frequent protective factors, especially *Sustained Presence,* which were particularly diminished due to the child domain of maternal parenting stress in PPD-mothers. These findings underscore the necessity of incorporating sociodemographic factors and child-related parenting stress into early prevention and intervention. Further prospective studies are needed to investigate the protective and risky nature of PEMA^TM^ factors for child development.

## Electronic supplementary material

Below is the link to the electronic supplementary material.

Supplementary Material 1

## Data Availability

The datasets generated and analyzed during the current study are not publicly available because they contain personal and sensitive material. Parental consent was obtained under the condition that the data would not be shared with third parties. This restriction was also required by the ethics committee; therefore, data cannot be made available.

## Abbreviations

BDI: Becks Depressions Inventar as the assessment of clinically relevant depressive symptoms within the last week
DERS: Difficulties in Emotion Regulation Scale as the assessment of clinically problems of negative emotion regulation
DSM-IV: Diagnostic and Statistical Manual of Mental Disorders, 4th edition according to the American Psychiatric Association
ICC: Intraclass correlation coefficients of reliability tests
MDD: Major Depressive Disorder
PEM: Parental Embodied Mentalizing as the nonverbal assessment of parental mentalizing capacity
PEMA™: Parental Embodied Mentalizing Assessment™ as the clinical adaption of the PEM to assess the embodied risk and protective factors of parental embodied mentalizing
PPD: Postpartum Depression, maternal mood disorder within the year after childbirth
PSI: Parenting Stress Index as the assessment of parenting stress in interaction with their child
SCID: Structured Clinical Interview for diagnoses of DSM-IV Axis I- and -II mental disorders
UKE: University Medical Center Hamburg-Eppendorf, Germany

## Footnotes

1 In the control group, four mothers showed “mild to moderate” and one mother “clinically relevant” depressive symptoms according to the BDI [39].
